# Prevalence and Clinical Significance of Occult Hepatitis B Infection in The Gambia, West Africa^[Author-notes jiab327-FM1]^

**DOI:** 10.1093/infdis/jiab327

**Published:** 2021-06-23

**Authors:** Gibril Ndow, Amie Cessay, Damien Cohen, Yusuke Shimakawa, Mindy L Gore, Saydiba Tamba, Sumantra Ghosh, Bakary Sanneh, Ignatius Baldeh, Ramou Njie, Umberto D’Alessandro, Maimuna Mendy, Mark Thursz, Isabelle Chemin, Maud Lemoine

**Affiliations:** Division of Digestive Diseases, Department of Metabolism, Digestion, and Reproduction, Imperial College London, London, United Kingdom; Disease Control and Elimination, Medical Research Council Unit The Gambia, London School of Hygiene and Tropical Medicine, Fajara, The Gambia; Disease Control and Elimination, Medical Research Council Unit The Gambia, London School of Hygiene and Tropical Medicine, Fajara, The Gambia; INSERM U1052, CNRS UMR5286, Center de Recherche en Cancérologie, Université Claude Bernard, Lyon, France; Unité D’Épidémiologie des Maladies Émergentes, Institut Pasteur, Paris, France; National Heart and Lung Institute, Faculty of Medicine, Imperial College London, London, United Kingdom; Edward Francis Small Teaching Hospital, Banjul, The Gambia; INSERM U1052, CNRS UMR5286, Center de Recherche en Cancérologie, Université Claude Bernard, Lyon, France; National Public Health Laboratories, Ministry of Health, Kotu, The Gambia; National Public Health Laboratories, Ministry of Health, Kotu, The Gambia; Edward Francis Small Teaching Hospital, Banjul, The Gambia; School of Medicine and Allied Health Sciences, University of The Gambia, Banjul, The Gambia; Disease Control and Elimination, Medical Research Council Unit The Gambia, London School of Hygiene and Tropical Medicine, Fajara, The Gambia; International Agency for Research on Cancer, World Health Organization, Lyon, France; Division of Digestive Diseases, Department of Metabolism, Digestion, and Reproduction, Imperial College London, London, United Kingdom; INSERM U1052, CNRS UMR5286, Center de Recherche en Cancérologie, Université Claude Bernard, Lyon, France; Division of Digestive Diseases, Department of Metabolism, Digestion, and Reproduction, Imperial College London, London, United Kingdom

**Keywords:** occult hepatitis B, prevalence, advanced liver disease, cirrhosis, hepatocellular carcinoma, Africa

## Abstract

**Background:**

Prevalence and clinical outcomes of occult hepatitis B infection (OBI) have been poorly studied in Africa.

**Methods:**

Using the PROLIFICA cohort, we compared the prevalence of OBI between hepatitis B surface antigen (HBsAg)-negative healthy adults screened from the general population (controls) and HBsAg-negative patients with advanced liver disease (cases), and estimated the population attributable fraction for the effect of OBI on advanced liver disease.

**Results:**

OBI prevalence was significantly higher among cases (15/82, 18.3%) than controls (31/330, 9.4%, *P* = .03). After adjusting for age, sex, and anti-hepatitis C virus (HCV) serology, OBI was significantly associated with advanced liver disease (odds ratio, 2.8; 95% confidence interval [CI], 1.3–6.0; *P* = .006). In HBsAg-negative people, the proportions of advanced liver disease cases attributable to OBI and HCV were estimated at 12.9% (95% CI, 7.5%–18.1%) and 16.9% (95% CI, 15.2%–18.6%), respectively.

**Conclusions:**

OBI is endemic and an independent risk factor for advanced liver disease in The Gambia, West Africa. This implies that HBsAg-negative people with liver disease should be systematically screened for OBI. Moreover, the impact of infant hepatitis B immunization to prevent end-stage liver disease might be higher than previous estimates based solely on HBsAg positivity.

## Teaser

This article reports a high prevalence of occult hepatitis B infection (OBI) in the general adult population in The Gambia, and shows that OBI is a risk factor for advanced liver disease accounting for more than 12% of cases.

Chronic infection with hepatitis B virus (HBV) affects approximately 250 million people globally and is a major cause of cirrhosis, hepatocellular carcinoma (HCC), and death [1]. By 2030 the World Health Organization (WHO) aims to reduce new HBV infections and HBV-related deaths by 90% and 65%, respectively [1]. These ambitious goals can only be achieved with sustained interruption of transmission and significant scale-up of HBV screen-and-treat interventions [2].

In resource-limited settings where HBV is highly endemic and nucleic acid testing is scarce, HBV screening relies on hepatitis B surface antigen (HBsAg) testing—either using serology or more often rapid point-of-care tests. HBsAg screening, however, fails to detect occult hepatitis B infection (OBI), which is characterized by either loss or repression of HBsAg, or HBV pre-S/S mutations [3, 4].

OBI is defined as presence of hepatic HBV DNA replication with or without detectable HBV DNA in blood with negative serum HBsAg detection [4]. Subjects with OBI, usually characterized by low HBV viremia <200 IU/mL [4, 5], could represent an important source of new HBV infections especially in blood transfusion services in sub-Saharan Africa where HBsAg testing is the primary screening tool for HBV and nucleic acid testing or hepatitis B core antibody (anti-HBc) screening are not routine [6]. In addition, subjects with OBI are at risk of developing HCC [7] and should benefit from regular screening for liver complications. Cirrhotic patients with undiagnosed OBI could miss potentially life-saving antiviral treatment opportunities [5].

To date, the prevalence of OBI in sub-Saharan Africa, its clinical significance in liver disease, and its public health impact on the HBV elimination goals remain unclear. Studies on OBI in sub-Saharan Africa are limited and mainly conducted in selected populations (HIV-infected adults [8–11], health care workers [12], blood donors [13, 14], and patients with HCC [15]) with reported prevalence ranging from less than 1% to more than 60%. However, the prevalence of OBI in the general population in sub-Saharan Africa remains poorly documented [16] and no studies so far have investigated the association between OBI and advanced liver disease in sub-Saharan Africa.

From large-scale community screenings in The Gambia [17, 18], we estimated the prevalence of OBI in the general adult population. To determine the clinical significance of OBI as a cause of advanced liver disease in The Gambia, we conducted a case-control study comparing healthy HBsAg-negative people from the community with HBsAg-negative adult patients with advanced liver disease [19], and then estimated the fraction of advanced liver disease attributable to OBI.

## METHODS

### Study Population

The Prevention of Liver Fibrosis and Cancer in Africa (PROLIFICA) program in The Gambia concurrently conducted 2 large studies: a community-based hepatitis B test-and-treat study [17] and a HCC case-control study (HC4) [18].

Between December 2011 and January 2014, the test-and-treat study [17] screened the general population living in 54 randomly selected enumeration areas (27 rural and 27 urban) in western Gambia. All inhabitants aged at least 30 years and living in each selected enumeration area were eligible and invited for HBsAg screening. From this screening intervention, we invited all HBsAg-positive participants, as well as about 1 in 10 HBsAg-negative participants from each enumeration area, for a full liver assessment. HBsAg-negative controls were randomly selected using statistical software (STATA 11, Stata Corporation). In addition, we also recruited 39 HBsAg-negative people identified through historical population-based serosurveys conducted in Keneba and Manduar villages in West Kiang District [18].

During the same period, the HC4 study [18] enrolled consecutive patients with suspected advanced liver disease referred to the outpatient liver clinic at the MRC unit The Gambia, which was the only liver clinic in the country at the time and received all suspected cases of liver disease. Therefore, participants enrolled in the test-and-treat and the HC4 studies are, respectively, representative of the general adult population and population with advanced liver disease in The Gambia.

At enrolment, all participants had standardized demographic, clinical, and laboratory investigations as previously described [17, 18]. All HBsAg-negative participants in both studies were included in this OBI study. Studies were approved by the MRC-Gambia Government Joint Ethics Committee and all participants signed an informed consent.

### OBI Case-Control Study

In the current analysis, cases were HBsAg-negative adults with advanced liver disease enrolled in the HC4 study [18] and controls were HBsAg-negative adults without liver disease enrolled from the community-based serosurveys [17, 18].

### Laboratory Analysis

#### DNA Extraction

DNA extractions from 200 µL plasma used either the QIAamp DNA Blood MiniKit (QIAgen) or the automated Arrow Nucleic Acid Extraction and Cell Separation Instrument (DiaSorin).

#### HBV Molecular Analysis

We used a nested polymerase chain reaction (PCR) assay (limit of detection [LOD] < 5 IU/mL) to detect HBV DNA in HBsAg-negative plasma. The nested PCR used previously published primers [19] to amplify 310 bps and 228 bp of the surface and polymerase regions of the HBV genome in the primary and nested reactions. A highly sensitive in-house quantitative PCR technique [20] was used to quantify HBV viremia in all samples with detectable HBV DNA on nested PCR.

#### HBV Serology

We confirmed negative HBsAg serology in all samples with detectable HBV DNA using a chemiluminescent microparticle immunoassay (Architect, Abbott; LOD, 0.1 IU/L) or the ultrasensitive Elecsys HBsAg II assay (Roche; LOD, 0.05 IU/L). All OBI-positive samples were tested for anti-HBc using an enzyme-linked immunosorbent assay (ELISA; Abnova).

#### Occult Hepatitis B Infection

OBI was defined as detectable HBV DNA in plasma with negative HBsAg serology. In a subanalysis, we further restricted the OBI definition to HBsAg-negative participants with HBV DNA levels <200 IU/mL (also called true OBI) because escape mutants in the S gene are associated with high-viremia OBI [4].

#### PreS/S Mutation Screening

In samples with detectable HBV DNA, we ran a second nested PCR targeting the preS/S region amplifying 315 bps and 113 bps in the primary and nested reactions, respectively (Supplemental Material).

#### Assessment of Liver Disease

To estimate liver fibrosis, liver stiffness measurement (LSM) was performed on fasted patients [21] using previously validated cutoff values (7.9 kPa and 9.5 kPa for fibrosis ≥F2 and F4, respectively) [22]. Abdominal ultrasound assessed features of cirrhosis, portal hypertension, and/or hepatic mass. HCC was diagnosed either histopathologically or clinically in patients who met at least 2 of the following: 1 or more hepatic mass(es) ≥2 cm consistent with HCC on abdominal ultrasound, α-fetoprotein ≥200 ng/mL, and cirrhosis.

### Statistical Analysis

Prevalence of OBI in the general population and among patients with advanced liver disease was estimated in HBsAg-negative participants enrolled in the community-based serosurveys [17, 18] and HC4 [19] studies, respectively. Categorical variables were compared using χ ^2^ test or exact test, and continuous variables were compared using Kruskal-Wallis test. To assess the clinical significance of OBI, we used logistic regression to determine the association between OBI and advanced liver disease in HBsAg-negative people. Potential confounders (age, sex, alcohol consumption [never vs ever], family history of liver cancer, positive hepatitis C virus [HCV], and human immunodeficiency virus [HIV] serologies) associated with advanced liver disease in univariable analysis (*P* < .05) were further included in multivariable logistic regression.

The population attributable fraction (PAF) and its 95% confidence interval (95% CI) were estimated for the effect of OBI and HCV on advanced liver disease [21]. All analyses were performed using STATA 13.1.

## RESULTS

### Prevalence of OBI in the General Population

Between December 2011 and January 2014, the test-and-treat study screened for HBsAg 5980 adults living in urban and rural communities representative of western Gambia [17] using the Determine rapid diagnostic kit, for which very good diagnostic performance was reported in The Gambia [23]. There were 5485 HBsAg-negative individuals; 479 of them were randomly selected and 305 accepted further clinical and virological assessment. Compared to HBsAg-negative adults who did not accept the invitation, those who accepted were more likely to be female (60.0% [183/305] vs 54.0% [94/174]; *P* = .2), and older (median age, 45 years; interquartile range [IQR], 36–57 versus 40 years; IQR, 34–53; *P* = .005).

**Figure 1.**
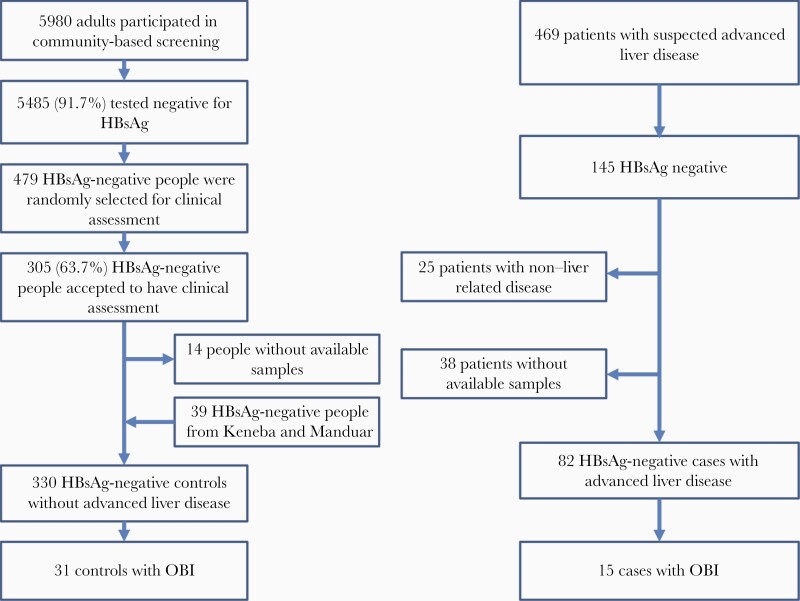
Study flow diagram.

After excluding 14 participants without an available sample and including 39 HBsAg-negative people from historical community-based screening [18], we finally had 330 HBsAg-negative controls without advanced liver disease (Figure 1). Thirty-one had detectable HBV DNA, giving an OBI prevalence of 9.4% (95% CI, 6.2–12.6). By restricting the analysis to those who had household member(s) tested for HBsAg (n = 259), we assessed whether having HBsAg-positive household member(s) was associated with OBI. There was no statistically significant association; the prevalence of OBI was 12.6% (11/87) and 8.7% (15/172) in those with and without HBsAg-positive household member(s), respectively (*P* = .3). When true OBI was defined as HBsAg-negative test and HBV DNA viral load <200 IU/mL [4], the prevalence was 2.7% (9/330).

### Prevalence of OBI Among Patients With Advanced Liver Disease

During the same period, the HC4 study [18] enrolled 469 patients with suspected advanced liver disease of whom 145 were HBsAg negative; 63 HBsAg-negative patients being excluded from this analysis (25 non-liver disease and 38 no samples available; Figure 1), we analyzed 82 HBsAg-negative patients with advanced liver disease (29 with fibrosis/cirrhosis and 53 with HCC; Figure 1).

The prevalence of OBI among all HBsAg-negative patients with advanced liver disease was 18.3% (15/82; 95% CI, 9.7%–26.8%). HBsAg-negative patients with significant fibrosis or cirrhosis but without HCC had the highest OBI prevalence (37.9%; 95% CI, 19.1%–56.7%). When true OBI (as defined by HBV viral load detectable but below 200 IU/mL) was considered, the prevalence of OBI among patients with advanced liver disease was 13.4% (11/82). We confirmed negative HBsAg serology in all OBI participants. The general characteristics of the HBsAg-negative participants are described in Table 1.

**Table 1.** General Characteristics of the Study Population

Characteristic	Healthy Controls From General Population (n = 330)	Cases with Advanced Liver Disease		*P* Value^a^
		Significant Liver Fibrosis and Cirrhosis (n = 29)	HCC (n = 53)	
Age, y, median (IQR)	46 (36–59)	53 (33–67)	56 (48–65)	.06
Male sex, No. (%)	129 (39)	20 (69)	38 (72)	<.001
BMI, median (IQR)	22.4 (19.8–26.0)	18.9 (15.6–22.4)	19.6 (17.3–22.1)	<.001
Alcohol, No. (%)	22 (7)	1 (4)	8 (15)	.05
Family history of liver cancer, No. (%)	6 (2)	0	2 (4)	.6
Positive HCV serology, No. (%)	9 (3)	4 (14)	11 (22)	<.001
Positive HIV serology, No. (%)	12 (4)	0	3 (6)	.6
ALT, IU/L, median (IQR)	22 (17–27)	46 (23–76)	52 (35–79)	<.001
AST, IU/L, median (IQR)	27 (22–32)	78 (42–139)	114 (73–264)	<.001
GGT, IU/L, median (IQR)	24 (18–34)	182 (54–434)	315 (139–516)	<.001
Platelet/mm^3^, median (IQR)	216 (172–268)	264 (161–385)	269 (186–369)	.01
LSM, kPa, median (IQR)	4.5 (3.7–5.5)	26.0 (8.8–66.4)	58.9 (27.0–75.0)	<.001
OBI, No. (%)	31 (9)	11 (38)	4 (8)	<.001

Abbreviations: ALT, alanine transaminase; AST, aspartate transaminase; BMI, body mass index; GGT, γ-glutamyl transferase; HCC, hepatocellular carcinoma; HCV, hepatitis C virus; IQR, interquartile range; LSM, liver stiffness measurement; OBI, occult hepatitis B infection.

^a^ *P* values were obtained using Kruskal-Wallis test for continuous variables and χ ^2^ test for categorical variables.

### Characteristics of Participants With OBI

Table 2 summarizes the characteristics of OBI participants. All 31 controls with OBI from the general population had normal hepatic investigation. Compared to healthy controls with OBI (n = 31), cases with OBI (n = 15) were predominantly males (73%, *P* = .03), had higher median LSM (13.0 kPa [IQR, 7.9–48.0] vs 4.7 kPa [4.3–6.0]; *P* < .001), had higher liver transaminases (ALT 45 IU/L [IQR, 24–89] vs 20 IU/L [IQR, 17–30], *P* < .001); AST 82 IU/L [IQR, 29–142] vs 27 IU/L [IQR, 25–31], *P* < .001), and had lower HBV DNA levels (2.7 log IU/mL [IQR, 2.1–3.4] vs 3.7 log IU/mL [IQR, 3.4–4.0], *P* = .08).

**Table 2.** Clinical Characteristics of People With OBI by Disease Status

Characteristic	Controls With OBI (n = 31)	Cases With OBI (n = 15)	*P* Value
Age, y, median (IQR)	44 (38–66)	53 (35–56)	.8
Sex			
Male, No. (%)	12 (39)	11 (73)	.03
Female, No. (%)	19 (61)	4 (27)	
BMI, kg/m^2^, median (IQR)	21.9 (20.3–23.4)	21.8 (19.6–22.8)	.5
Alcohol, No. (%)			
Never	28 (93)	14 (100)	1.0
Ever	2 (7)	0	
Family history of liver disease, No. (%)			
No	31 (100)	15 (100)	NA
Yes	0	0	
ALT, IU/L, median (IQR)	20 (17–30)	45 (24–89)	<.001
AST, IU/L, median (IQR)	27 (25–31)	82 (29–142)	<.001
GGT, IU/L, median (IQR)	21 (15–28)	199 (24–354)	<.001
Platelet/mm^3^, median (IQR)	203 (174–245)	256 (186–305)	.2
LSM, kPa, median (IQR)	4.7 (4.3–6.0)	13.0 (7.9–48.0)	<.001
HBV DNA, log IU/mL, median (IQR)^a^	3.7 (3.4–4.0)	2.7 (2.1–3.4)	.08
HBV DNA level, IU/mL, No. (%)			
<50	7 (23)	9 (73)	.003
50–2000	5 (16)	4 (7)	
2000–20 000	18 (58)	1 (7)	
>20 000	1 (3)	1 (7)	
Quantitative HBsAg level, IU/mL, No.			
>0.05	NA	0	NA
<0.05	NA	15	
Anti-HBc serology, No. (%)			
Negative	2 (6)	0	
Positive	29 (94)	15 (100)	
Anti-HCV serology, No. (%)			
Negative	31 (100)	13 (87)	.1
Positive	0	2 (13)	
Anti-HIV serology, No. (%)			
Negative	30 (97)	15 (100)	1.0
Positive	1 (3)	0	
HBV genotype, No. (%)			
A	0 (0)	5 (36)	NA
E	31 (100)	9 (64)	
Pre-S2 mutation, No. (%)			
Wild type	26 (84)	11 (79)	.7
Mutation	5 (16)	3 (21)	

Abbreviations: ALT, alanine transaminase; AST, aspartate transaminase; BMI, body mass index; GGT, γ-glutamyl transferase; HBc, hepatitis B core; HBsAg, hepatitis B surface antigen; HBV, hepatitis B virus; HCC, hepatocellular carcinoma; HCV, hepatitis C virus; HIV, human immunodeficiency virus; IQR, interquartile range; LSM, liver stiffness measurement; OBI, occult hepatitis B infection.

^a^After excluding those with detectable, but not quantifiable, HBV DNA.

Pre-S2 deletions were detected in 18% (8/45) of adults with OBI, with no significant difference between controls with OBI (5/31) and cases with OBI (3/14; *P* = .7). All community-screened controls with OBI were infected with HBV genotype E. In contrast, all 4 HCC cases with OBI were infected with genotype A. There was no statistically significant difference in HCV and HIV prevalence between cases with OBI and controls with OBI. In 330 community-screened participants, there were no clinical or demographic differences between those with and without OBI (Table 3).

**Table 3.** General Characteristics of Community-Screened Adults With and Without OBI

Characteristic	HBsAg-Negative Adults From General Population (n = 330)		
	OBI (n = 31)	No OBI (n = 299)	*P* Value
Age, median (IQR)	44 (38–66)	46 (36–58)	.6
Sex, No. (%)			
Male	12 (39)	117 (39)	.9
Female	19 (61)	182 (61)	
BMI, median (IQR)	21.9 (20.3–23.4)	22.5 (19.8–26.3)	.2
Alcohol, No. (%)			
Never	28 (93)	277 (93)	.9
Ever	2 (7)	20 (7)	
Family history, No. (%)			
No	31 (100)	293 (98)	.4
Yes	0	6 (2)	
Anti-HCV, No. (%)			
Negative	31 (100)	289 (97)	.3
Positive	0	9 (3)	
Anti-HIV, No. (%)			
Negative	30 (97)	287 (96)	.9
Positive	1 (3)	11 (4)	

Abbreviations: BMI, body mass index; HCV, hepatitis C virus; HIV, human immunodeficiency virus; IQR, interquartile range; OBI, occult hepatitis B infection.

### OBI is an Independent Risk Factor of Advanced Liver Disease

OBI prevalence was significantly higher among cases with advanced liver disease (18.3%; 95% CI, 9.7%–26.8%) compared to the control group from the general population (9.4%; 95% CI, 6.2%–12.6%; *P* = .03; Table 4). In addition to OBI, older age group (≥45 years), male sex, and positive anti-HCV antibody were also associated with advanced liver disease in HBsAg-negative people in univariable analysis. In multivariable analysis, all except age remained significantly associated with advanced liver disease (Table 4): male sex (odds ratio [OR], 1.6; 95% CI, 2.5–8.4; *P* < .001), positive anti-HCV antibody (OR, 10.8; 95% CI, 4.0–29.2; *P* < .001), and OBI (OR, 2.8; 95% CI, 1.3–6.0; *P* = .006).

**Table 4.** Risk Factors for Advanced Liver Disease in HBsAg-Negative Adults

Variables	Cases With Advanced Liver Disease, No. (%) (n = 82)	Healthy Controls From General Population, No. (%) (n = 330)	Crude OR		Adjusted OR^a^	
			OR (95% CI)	*P* Value	OR (95% CI)	*P* Value
Age, y						
<45	23 (29)	150 (45)	1.0	.01	1.0	.1
≥45	55 (71)	180 (55)	2.0 (1.2–3.4)		1.6 (.9–2.8)	
Sex						
Female	24 (29)	201 (61)	1.0	<.001	1.0	<.001
Male	58(71)	129 (39)	3.8 (2.2–6.4)		1.6 (2.5–8.4)	
Alcohol						
Never	71 (89)	305 (93)	1.0	.2		
Ever	9 (11)	22 (7)	1.8 (.8–4.0)			
Family history						
No	80 (98)	324 (98)	1.0	.7		
Yes	2 (2)	6 (2)	1.4 (.3–6.8)			
Anti-HCV						
Negative	64 (81)	320 (97)	1.0	<.001	1.0	<.001
Positive	15 (19)	9 (3)	8.3 (3.5–19.9)		10.8 (4.0–29.2)	
Anti-HIV						
Negative	78 (96)	317 (96)	1.0	.9		
Positive	3 (4)	12 (4)	1.0 (.3–3.7)			
OBI						
Negative	67 (82)	299 (91)	1.0	.03	1.0	.006
Positive	15 (18)	31 (9)	2.2 (1.1–4.2)		2.8 (1.3–6.0)	

Abbreviations: CI, confidence interval; HBsAg, hepatitis B surface antigen; HCV, hepatitis C virus; HIV, human immunodeficiency virus; OBI, occult hepatitis B infection; OR, odds ratio.

^a^Model included age, sex, anti-HCV, and OBI.

In HBsAg-negative people, the proportion of advanced liver disease cases attributable to OBI (PAF) was estimated at 12.9% (95% CI, 7.5%–18.1%). The PAF for HCV was 16.9% (95% CI, 15.2%–18.6%).

## Discussion

Our study reports a high prevalence of OBI in both the general population (9.4%) and among patients with advanced liver disease (18.3%), suggesting that OBI is an independent risk factor for advanced liver disease in The Gambia, West Africa. OBI prevalence has been poorly reported in the general population in sub-Saharan Africa. Most studies on OBI in Africa were conducted in selected populations. Using community-based screenings, OBI prevalence in the HBsAg-negative general adult population was high (9.4%), similar to the HBsAg prevalence (8.5%) previously reported by our group [17]. This finding raises an important public health concern because current hepatitis B screening strategies in resource-limited countries are based on HBsAg serology alone and thus cannot identify individuals with OBI.

In Uganda, the prevalence of OBI among an urban population screened in hospital in Kampala was 30% [16]. Studies conducted in HIV-infected individuals in Sudan and Mozambique also reported high OBI prevalence of 8.3% and 17.5%, respectively [8, 10] In Nigeria, OBI was found in 17% of blood donors [14].

In our study, we were unable to identify predictors of OBI among community-screened healthy adults. From a public health perspective, the absence of associated factors with OBI among the general population makes it difficult to develop targeted interventions for the identification of OBI. Importantly, age did not differ between community-screened healthy controls with or without OBI, suggesting that OBI might not only be accounted for by HBsAg loss in the ultimate phase of the natural history of chronic hepatitis B infection.

To the best of our knowledge, the clinical outcomes of OBI in Africa have been poorly assessed. Using biochemical markers of liver fibrosis, Carimo et al found that HIV-infected subjects with OBI are at higher risk of liver fibrosis than HIV-infected subjects without HBV infection [10]. However, the clinical impact of OBI in non-HIV population exposed to the African environment is unknown.

Our study found that OBI is more frequently observed in cases with advanced liver disease (18.3%) compared to community-screened healthy controls (9.4%). Interestingly, patients with HCC had a lower prevalence of OBI than patients with significant fibrosis or cirrhosis (8% vs 38%). This might reflect the natural history of hepatitis B in Africa where HCC is often observed without cirrhosis, therefore in an earlier phase in the natural history of chronic HBV infection before the spontaneous loss of HBsAg. A direct role of HBV genotype A, mainly associated with HCC in our population [18], or aflatoxin exposure remains to be elucidated. A prospective analysis with serial sample analysis of a larger longitudinal cohort of participants with OBI may find answers.

Importantly, our study found that OBI is an independent risk factor for advanced liver disease (OR, 2.8; 95% CI, 1.3–6.0; *P* = .006) in The Gambia. Moreover, OBI remained an independent factor of advanced liver diseases even when the OBI definition was restricted to patients with HBV viral load <200 IU/mL (OR, 7.0; 95% CI, 2.5–20.0; *P* < .001). This is in line with a previous meta-analysis reporting a 2.9-fold increased risk of HCC in individuals with OBI [7], with increased relative risk of HCC in both HCV and non-HCV–infected populations. This meta-analysis included 16 studies (3256 subjects), mainly from Asia, but none from Africa. Our study, therefore, provides additional evidence on the association between OBI and liver complications in Africa.

Further, our study found that 12.9% (95% CI, 7.5–18.1) of advanced liver disease cases in HBsAg-negative people in The Gambia were attributable to OBI. Therefore, OBI is responsible for a significant proportion of advanced liver disease cases, further increasing the population attributable risk for HBV-related advanced liver disease in The Gambia. This finding implies that the impact of infant hepatitis B immunization in preventing HBV infection, and by extension reducing the incidence of HBV-related liver disease, may be much higher than previous estimates based on PAF using HBsAg positivity alone [24].

Our findings could have important clinical implications in the management of significant liver disease and HCC screening in Africa. Patients with cirrhosis and OBI would be eligible for antiviral therapy. Antiviral therapy for HBV using tenofovir is becoming more accessible in many resource-limited countries at very low cost (US$ 1–2/month). It is therefore urgent to improve the identification of HBV cases including subjects with OBI-attributable advanced liver disease who would otherwise miss opportunities to receive potentially life-saving treatment. Therefore, our study suggests that in Africa, HBsAg-negative subjects with advanced liver disease and/or HCC should be systematically tested for HBV DNA. Current screening strategies for HBV do not systematically include anti-HBc serology or HBV DNA measurement due to cost and logistical constraints. In addition, our results suggest that OBI subjects should be regularly screened for advanced liver disease and HCC. Both approaches imply additional costs for HBV-endemic countries where OBI may be highly prevalent and resources for these additional screenings severely limited.

The mechanisms of OBI and its impact on liver disease remain debated. OBI is often considered as the ultimate phase of the natural history of chronic hepatitis B following HBsAg loss [4]. However, the similar age between healthy adults with and without OBI in our study does not support this hypothesis.

HBsAg variants resulting from mutations in the S gene may result in poor detection of HBsAg using commercially available serology assays [4]. In our study, only a minority of participants with OBI (16% of controls and 21% of cases) had detectable pre-S mutations, with no difference in HBsAg mutations according to liver disease severity. As a result, viral mutation cannot fully explain the high OBI prevalence and its relationship with advanced liver disease and HCC. In our experience, pre-S2 deletion mutants are only observed in coinfection with wild-type HBV able to produce HBsAg [25]. The impact of OBI on HCC development is certainly related to direct liver carcinogenesis through the integration of HBV DNA into the host genome leading to the synthesis of pro-oncogenic proteins [5].

Our study has some limitations. First, we analyzed samples collected at a single time point and did not perform liver biopsies to measure hepatic covalently closed circular DNA levels. HBV DNA in OBI is only detected intermittently in blood [4] therefore analyzing serial samples increases diagnostic accuracy and might even increase the estimated OBI prevalence. Liver biopsies are difficult to perform in sub-Saharan Africa and their indication difficult to justify in healthy controls. Moreover, there are no widely available standards for detecting OBI in liver tissue [4]. Second, we did not assess the long-term clinical impact of OBI in our study population. In particular, the rate of liver disease progression and loss of HBV DNA in patients with OBI in Africa is currently unknown. We will address this question in a longitudinal study. Third, we were unable to measure HCV RNA in the 2 participants with OBI who had positive HCV serology. Fourth, we did not assess the role of aflatoxin exposure on liver disease severity in individuals with OBI, nor the prevalence of HBsAg-immune complexes. As a result, the mechanisms underlying the development of OBI and its impact on liver outcomes need to be further elucidated. Finally, we did not characterize alcohol use in detail. Alcohol use is rare in our cohort, and alcohol has not been associated with advanced liver disease in this cohort, neither in this study nor previous publications, possibly due to the difficulty in ascertaining its consumption in its cultural/religious context [18, 19, 23].

In conclusion, OBI is frequently observed in The Gambia and is an independent risk factor for liver fibrosis, cirrhosis, and HCC accounting for 12.9% of advanced liver disease cases in HBsAg-negative adults. Strategies to identify adults with OBI need to be developed in Africa. Access to low-cost HBV DNA or reliable alternatives to HBV nucleic acid testing is urgent. Our findings could have important clinical, public health, and economic implications on HBV and liver disease management in Africa, as well as on the 2030 WHO objectives for HBV elimination.

## Supplementary Data

Supplementary materials are available at *The Journal of Infectious Diseases* online. Consisting of data provided by the authors to benefit the reader, the posted materials are not copyedited and are the sole responsibility of the authors, so questions or comments should be addressed to the corresponding author.

jiab327_suppl_Supplementary_Materials

## Notes

***Acknowledgments.*** We are grateful to the Medical Research Council (MRC) Unit The Gambia at London School of Hygiene and Tropical Medicine, the Gambian Ministry of Health and Social Welfare, and the National Public Health Laboratory The Gambia for hosting and supporting the PROLIFICA programme. We are also grateful to Gilead US company for its long support to the PROLIFICA program. We thank all study participants, the PROLIFICA and Gambia Hepatitis Intervention Study teams in The Gambia, and the MRC unit The Gambia data management team.

***Author contributions.*** G. N., Y. S., M. T., and M. L. designed the study, analyzed the data, and drafted the manuscript. G. N., A. C., D. C., M. L. G., and S. G. conducted the experiments, and Y. S. was in charge of statistical analysis. G. N., Y. S., S. T., B. S., I. B., R. N., U. D. A., M. M., M. T., I. C., and M. L. were responsible for the study cohorts and samples. All authors reviewed and approved the final version.

***Financial support.*** This work was supported by the European Commission (grant number FP7–AFRICA–2010; Collaborative Project PROLIFICA (grant number 265994); and National Institute for Health Research Imperial Biomedical Research Centre. G. N. is supported by a Gilead Public Health award. D. C. and I. C. are supported by ANRS (Agence Nationale de Recherches sur le Sida et les hépatities virales). M. L. holds a UK Medical Research Council new investigator award.
