# Supplementary material for: Prevalence and Clinical Significance of Occult Hepatitis B Infection in The Gambia, West Africa
Source: J Infect Dis. 2021 Jun 23;226(5):862–70. doi: 10.1093/infdis/jiab327 (PMC9470103; doi:10.1093/infdis/jiab327)
Supplement: jiab327_suppl_Supplementary_Materials [file jiab327_suppl_supplementary_materials.docx]

**PreS/S mutation screening**

In samples with detectable HBV DNA, we ran a second nested PCR targeting the preS/S region amplifying 315bps and 113bps in the primary and nested reactions respectively using Q5 High-Fidelity DNA polymerase (New England BioLabs) and primers 315S-fwd (5’-CCAGCAGATCCTCCTCCTGC-3’), 315AS-rev (5’-AAACCCCGCCTGTAACACGAG-3’), 113S-fwd (5’-ATTCCACAACATTCCACCAAGC-3’) and 113AS-rev (5’-GAGTGAGGCAGTAGTCGGAAC-3’). Sequencing of the purified PCR product was performed by Genewiz, and mutations and genotypes analysed using the NCBI Genotyping tool using Q5 High-Fidelity DNA polymerase (New England BioLabs) and primers 315S-fwd (5’-CCAGCAGATCCTCCTCCTGC-3’), 315AS-rev (5’-AAACCCCGCCTGTAACACGAG-3’), 113S-fwd (5’-ATTCCACAACATTCCACCAAGC-3’) and 113AS-rev (5’-GAGTGAGGCAGTAGTCGGAAC-3’). Sequencing of the purified PCR product was performed by Genewiz, and mutations and genotypes analysed using the NCBI Genotyping tool.

*Ref: Rozanov M, Plikat U, Chappey C, Kochergin A, Tatusova T. A web-based genotyping resource for viral sequences. Nucleic Acids Res. 2004;32 (suppl_2): W654–659*
